# Characterization of genetic changes associated with daptomycin nonsusceptibility in *Staphylococcus aureus*

**DOI:** 10.1371/journal.pone.0198366

**Published:** 2018-06-07

**Authors:** Zhuo Ma, Erica Lasek-Nesselquist, Jackson Lu, Ryan Schneider, Riddhi Shah, George Oliva, Janice Pata, Kathleen McDonough, Manjunath P. Pai, Warren E. Rose, George Sakoulas, Meenakshi Malik

**Affiliations:** 1 Albany College of Pharmacy and Health Sciences, Albany, New York, United States of America; 2 Wadsworth Center, New York State Department of Health, Albany, New York, United States of America; 3 Universtiy of Wisconsin-Madison, School of Pharmacy, Madison, Wisconsin, United States of America; 4 Center for Immunity, Infection & Inflammation, UCSD School of Medicine, La Jolla, California, United States of America; Leibniz-Institute DSMZ, GERMANY

## Abstract

The extensive use of daptomycin (DAP) for treatment of methicillin-resistant *Staphylococcus aureus* (MRSA) infections in the last decade has led to the emergence of DAP non-susceptible (DNS) *Staphylococcus aureus* strains. A better understanding of the molecular changes underlying DAP-non-susceptibility is required for early diagnosis and intervention with alternate combination therapies. The phenotypic changes associated with DNS strains have been well established. However, the genotypic changes—especially the kinetics of expression of the genes responsible for DAP-non-susceptibility are not well understood. In this study, we used three clinically derived isogenic pairs of DAP-susceptible (DAP-S) and DNS *S*. *aureus* strains to study gene expression profiles with the objective of identifying the potential genotypic changes associated with DAP-nonsusceptibility. We determined the expression profiles of genes involved in cell membrane (CM) charge, autolysis, cell wall (CW) synthesis, and penicillin binding proteins in DAP-S and DNS isogenic pairs. Our results demonstrate characteristic expression profiles for *mprF*, *dltABCD*, *vraS*, *femB*, and *pbp2a* genes, which are common to all the DNS *S*. *aureus* strains tested. Whole genome sequencing of DAP-S and DNS clinical isolates of *S*. *aureus* showed non-synonymous mutations in all DNS strains in genes involved in CM charge, CM composition, CW thickness and CW composition. To conclude, this study unravels some of the complex molecular changes involved in the development of DAP-nonsusceptibility by demonstrating distinct differences in gene expression profiles and mutations in the DNS *S*. *aureus* strains. This knowledge will aid in rapid identification of DNS *S*. *aureus* in clinical settings.

## Introduction

The past decade has seen a steep rise in antibiotic resistance amongst Gram-positive bacterial pathogens including *Staphylococcus aureus*. Reports indicate that 95% of the clinical isolates of *S*. *aureus* in the USA are penicillin resistant and more than 50% are methicillin resistant (MRSA) [1]. In addition to MRSA, the antibiotic resistance scenario is further complicated by the emergence of vancomycin-resistant *S*. *aureus* (VRSA) and vancomycin-intermediate *S*. *aureus* (VISA) strains [2]. As an effective alternative, daptomycin (DAP) was approved for clinical use in the USA in 2003 for treatment of infections caused by methicillin-susceptible (MSSA), MRSA, and VISA strains. In 2006, DAP was approved for the treatment of bacteremia and right sided endocarditis caused by MSSA and MRSA [3]. DAP is a cyclic lipopeptide antibiotic that shows excellent activity against a variety of Gram-positive bacteria. DAP binds irreversibly to the bacterial cell membrane (CM) in a calcium-dependent manner, and cause cell death by disruption and depolarization of CM [4, 5]. DAP also inhibits biosynthesis of cell wall (CW) components such as lipoteichoic acid and interferes with bacterial cell division [6]. In clinical settings; prolonged underdosing of DAP, ineffective penetration of DAP due to infective endocarditis or osteomyelitis, and previous exposure to antimicrobial peptides or glycopeptide antimicrobials have resulted in emergence of DAP non-susceptible (DNS) *S*. *aureus* strains [7–10].

Extensive use of DAP for treatment of infections caused by MRSA and VISA has resulted in evolution of DNS *S*. *aureus* strains [11–15]. The DAP-nonsusceptibility is associated with several phenotypic as well as genotypic changes [16]. The phenotypic changes in DNS *S*. *aureus* strains include enhanced positive cell surface charge [11, 17] due to increase in positively charged lysyl-phosphatidyl glycerol (L-PG) phospholipids [18, 19], increased CW thickness due to increased teichoic acid synthesis, and altered CM fluidity due to changes in the fatty acid composition [4, 20, 21]. These phenotypic changes result from single or multiple genotypic changes. Mutation and increased transcription of the *mprF* gene; an L-PG synthase results in increased production of L-PG and enhancement of the net cell surface positive charge [22–24]. Mutations in the *dltABCD* operon similar to *mprF* result in increased CM positive charge [25]. Mutation of *walK* gene that encodes a histidine kinase sensor [18, 26] and *csl2*; a cardiolipin synthase [27] alter CW metabolism, cell permeability [28], and cause accumulation of phosphotidyl glycerol [29], respectively. These changes eventually contribute to the development of DNS *S*. *aureus*. Taken together, these studies have established the phenotypic and genetic changes associated with DNS in *S*. *aureus*. However, the kinetics and alterations in the expression of genes responsible for causing these changes have not been well characterized.

In this study, we evaluated the expression profile of genes involved in CM charge, autolysis, CW synthesis and penicillin binding proteins using three DAP-S/DNS isogenic pairs A6300/A6298, R6837/R6838 JH1/JH4-JH5 isolated from patients. These strains are derivative of a major MRSA clone USA100. The A6300/A6298 isogenic pair was isolated from a case of bacteremia and prosthetic joint infection in a hospital in Massachusetts. A6300 is a MRSA strain that later developed heteroresistance to vancomycin during vancomycin therapy and a small increase in minimum inhibitory concentration (MIC) to DAP into a non-susceptible range, and was designated as A6298 [30]. The isogenic pairs R6837/R6838 originally described as D592/D712, were isolated from a patient with prolonged bacteremia secondary to osteomyelitis isolated at Westchester Medical Center in Valhalla, New York. The parent R6837 (D592) strain is DAP-S, MRSA and hVISA strain that subsequently mutated to R6838 (D712) as DNS, MRSA, VISA following a 20-day period of vancomycin and DAP therapy [31, 32]. JH1 and JH4 are among the series of isolates recovered from a congenital heart disease patient following treatment failure with vancomycin, rifampin and imipenem in Baltimore, MD [33]. JH1 recovered before the beginning of DAP antibiotic therapy is fully sensitive to vancomycin, while the subsequent isolates from the same patient recovered after the initiation of vancomycin therapy showed decreased susceptibility to vancomycin as well as DAP. The MIC of JH1 for DAP was 0.01μg/mL, while it increased to 0.05μg/mL for JH2 and subsequent isolates [34]. Although related genetically, the clinical isolates selected for this study are very distinct clinically and microbiologically. The development of DAP-nonsusceptibility in these strains occurred as separate events. In A6298 and JH4 DNS strains, the DAP-nonsusceptibility occurred even before DAP was introduced for clinical use. On the other hand, R6838 developed into a DNS *S*. *aureus* following a 3-week exposure to DAP. In this study, we investigated the expression profile of genes and mutations associated with DAP-nonsusceptibility by whole genome sequencing to identify genotypic changes that can serve as markers for differentiation of DAP-S and DNS clinical isolates.

## Material and methods

### Bacterial strains and antibiotic

Isogenic pairs of susceptible parent strains of DAP-S and DNS A6300/A6298 isolated from a patient suffering from bacteremia and prosthetic joint infection in a hospital in Massachusetts [30], R6837/R6838 isolated from a patient at Westchester Medical Center, New York [31], and JH1/JH4 isogenic pair recovered from a congenital heart disease patient following treatment failure with vancomycin rifampin and imipenem at Johns Hopkins in Baltimore, MD [34]. The reference *S*. *aureus* subsp. *aureus* Rosenbach strain (ATCC^®^ 29213™) was obtained from BEI Resources, Manassas, VA.

Clinical grade Cubicin^®^ (injectable daptomycin) was purchased from Albany Medical Center Outpatient Pharmacy, Albany, NY. The DAP was supplied as 500 mg vials and was reconstituted in sterile water to achieve desired concentrations to be used in various experiments.

### Bacterial growth curves

Isogenic DAP-S and DNS pairs A6300/A6298, R6837/R6838 and JH1/JH4, JH5 of *S*. *aureus* were streaked on Trypticase™ Soy Agar II with 5% sheep blood (BD Biosciences) using sterile loops and incubated overnight at 37°C with 5% CO_2_. The colonies were resuspended in 10mL of Muller Hinton Broth (BD BBL^TM^) and the optical density (OD_600_) was adjusted at ~0.01. The cultures were grown in a shaking incubator at 37°C. Aliquots were collected at 1 hour intervals for a period of 8 hours. The aliquots were diluted 10-fold and plated on sheep blood agar plates to quantify bacterial numbers. The plates were incubated at 37°C overnight. The colonies were counted and the results were expressed as Log_10_ CFU/mL.

### Antimicrobial susceptibility studies

The minimum inhibitory concentration (MIC) values were determined for each DAP-S/DNS *S*. *aureus* isogenic pair using either Epsilometer test (E-test) or broth microdilution method. The MIC was considered to be the lowest concentration of the drug required to inhibit bacterial growth. All susceptibility testing was performed in triplicate and was repeated at least twice. For E-test cation-adjusted Muller-Hinton Broth (CAMHB) containing 25mg/mL calcium and 12.5 mg/mL magnesium was used to grow isogenic pairs of DAP-S and DNS *S*. *aureus* strains to an OD_600_ of 0.08–0.09 in 5 mL tubes. Sterile cotton swabs were used to uniformly streak a lawn of each clinical isolate on trypticase soy agar plates containing 5% sheep blood. The cultures were allowed to be completely adsorbed. Using sterile tweezers, the E-test strips containing varying concentrations of DAP (BioMérieux Inc.) were place on the streaked agar plates. The plates were incubated at 37°C for 18–20 hours. The elliptical zones of inhibitions were read to determine the MIC.

The broth dilution method was performed according to Clinical Laboratory Standards Institute (CLSI) guidelines. Blood agar plates streaked with isogenic pairs of DAP-S and DNS *S*. *aureus* strains and a control ATCC® 29213 were grown overnight. Single colonies were picked, inoculated in CAMHB and grown to achieve an OD range of 0.08–0.09 in 5-mL which corresponds to 10^8^ CFU/mL. DAP was diluted to a starting concentration of 32 μg/mL of DAP and then diluted two-fold in a sterile 96-well plate to achieve a final concentration of 8, 4, 2, 1, 0.5, 0.25, and 0 μg/mL. In accordance with CLSI standards, bacterial suspension diluted to a final concentration of 5.5x10^5^ CFU/mL was added to each well of the plate containing varying concentrations of DAP. After the drug and bacterial inoculum were added, the 96-well plate was incubated for 24 hours and read.

### Quantitative real-time polymerase chain reaction (qRT-PCR)

DAP-S and DNS strains grown to a mid-logarithmic phase in MHB were used in all the experiments. The aliquots were collected after 0, 2, 6, and 24 hours of growth for RNA isolation. Isolation of RNA was carried out using the RNA isolation PureLink kit (Invitrogen) according to the manufacturer’s instructions. Two-step qRT-PCR was carried out by first generating the cDNA using the BioRad® iScript™ cDNA kit and then performing the qRT-PCR assay using the BioRad iQSYBR Green Supermix kit. The primer sequences used for transcriptional analysis of genes are shown in Table 1. Normalization of the target gene was done using 16S rRNA as an internal control.

**Table 1:** List of primer sequences used for transcriptional analysis.

Gene	Primer	Sequence
*mprF*	Forward	5’ TTA TAG GTT TCG GTG GCT TT 3’
	Reverse	5’ GAT GCA TCG AAA ACA TGG AA 3’
*dltA*	Forward	5’ TAA CCA AGC GCC ATT TTC AT 3’
	Reverse	5’ AAC GGC TCA CTA ACG CTT TT 3’
*dltB*	Forward	5’ GCC ATT AGC ACT TGT GAA AGT GTT 3’
	Reverse	5’ TCC AGA TGA AAT CGT TGG GA 3’
*dltC*	Forward	5’ CCA GAC GTA GAA ATT TTT GAA GAA 3’
	Reverse	5’ CGT AAC TCT TCT AAT GCT TCA ACG 3’
*dltD*	Forward	5’ GCA TTA AAT AAG CAT AAC GCC AAC 3’
	Reverse	5’ GAC ATG TTT TTC TGC TGG AAC A 3’
*vraSR*	Forward	5’ TGC TTA CAG AAC GAG AAA TGG AAA 3’
	Reverse	5’ CGT TTT AAT AGT CGA TGC A 3’
*femA*	Forward	5’ GAT TCC ATA AAG GAT TTG ATC CTG 3’
	Reverse	5’ AAG GTA CTA ACA CAC GGT CTT TG 3’
*femB*	Forward	5’ CCT TGA AGG TAA AAC ACC CGA 3’
	Reverse	5’ GTC ATT CAA TTC CTG TTG CAA CT 3’
*walKR*	Forward	5’ ACT TTG GCG ATG TAC GTA CG 3’
	Reverse	5’ AGC CCG ATA ATT TGC ATA CC 3’
*Atl*	Forward	5’ ACA ACG CAC GGA TTA CAC ATC T 3’
	Reverse	5’ CCG ATA AAC ATT GAC ATC TTG C 3’
*lytM*	Forward	5’ CGA GTC AAA GCC AAC AGC ATA T 3’
	Reverse	5’ TTT CAG GCA TTG CAT AGT CG 3’
*Pbp2a*	Forward	5’ TTT TTG CCA ACC TTT ACC ATC G 3’
	Reverse	5’ TAC TGC TAT CCA CCC TCA AACA 3’
*Pbp1*	Forward	5’ AGG TAG CGG TTT TGT GTC C 3’
	Reverse	5’ TAT CCT TGT CAG TTT TAC TGT C 3’

### Transmission electron microscopy (TEM)

TEM was conducted using a previously published protocol. A Philips CM120 electron microscope with Image 1.39t software was used to measure and analyze CW thickness and differences in septum formation between DAP-S and DNS *S*. *aureus* clinical isolates. Each strain was streaked on blood agar and incubated overnight at 37°C. The following day, 1–2 colonies from the incubated plates were inoculated in MHB and incubated overnight. The bacterial cultures were centrifuged and the pellets were washed with cold sodium phosphate buffer. The pellets were fixed in electron microscopy buffer, post-fixed in 1% osmium tetroxide, dehydrated and embedded in Spurr’s epoxy resin. Ultrathin sections of the samples were examined using the electron microscope. CW thickness was measured from each quadrant of the cell on a minimum of 25 cells.

### CM fluidity assay

Membrane fluidity of DAP-S and DNS clinical isolates was determined spectroflurometrically using a fluorescent probe, 1,6-diphenyl-1,3,5-hexatriene (DPH) as described previously [20]. Briefly, the overnight cultures were inoculated in fresh MHB medium and grown to an OD_600_ between 0.2–0.5. Then, bacterial cells were pelleted, washed with normal saline (0.85% NaCl) and resuspended to an OD_600_ of 0.3 in normal saline (0.85% NaCl) containing 2μM DPH. The DPH suspension was incubated for an hour at 37°C and transferred to preheated cuvettes. The fluorescence was determined in an ISS Koala spectrofluorometer with a temperature-controlled cuvette holder maintained at 37°C. The excitation and emission wavelengths for DPH were 360 nm and 426 nm, respectively. The polarization index (*p*) of each sample was calculated and recorded as described previously [20]. Lower polarization index value is an indicator of higher degree of cell membrane fluidity [35].

### Cytochrome C binding assay

Cytochrome C binding assay was carried out as described previously [19, 36, 37]. Briefly, bacterial cells were grown for 24 hours in MHB media and washed twice with 20 mM MOPs buffer (pH 7.0). The cells were adjusted to a final OD_600_ of 0.150 in the MOPs buffer and then incubated with 0.5mg/mL cytochrome C at room temperature for 10 minutes in a total volume of 500 μl. The reaction mixture was centrifuged for 5 minutes and the amount of the unbound cytochrome C was quantified spectrophotometrically at 530nm in the supernatants.

### Whole genome sequencing

Overnight cultures of DAP-S and DNS *S*. *aureus* clinical isolates were treated with lysostaphin and lysozyme to disrupt the peptidoglycan layer of the CW, genomic DNA isolated using PureLink Genomic DNA Mini Kit (Invitrogen), and submitted to the Wadsworth sequencing core. Whole genome sequencing libraries were prepared with the Nextera DNA library preparation kit (Illumina) and sequenced using the standard 500 cycle V2 protocol on an Illumina MiSeq. Whole genome sequences were required to have an average coverage of at least 80x for the genome before analysis.

The subroutine bbduk from bbtools v36.38 (https://sourceforge.net/projects/bbmap)) quality trimmed raw Illumina reads and removed any remaining adaptors/primers with the following parameters: qin = 33, ktrim = r, mink = 11, trimq = 20, minlength = 100, tbp = t, and tpe = t. BWA v.0.7.5 [38] with the “mem” option aligned processed reads for all isogenic pairs to the N315 genome (BA000018.3). Additionally, reads from JH4 and JH5 resistant strains were aligned to their JH1 parental genome (RefSeq accession number GCF_000017125.1) and reads from A6298 were aligned to the susceptible parental A6300 genome (RefSeq accession number GCF_000174515.1). Because the R6837/R6838 clinical pair lacked a representative genome in GenBank, we aligned R6838 reads to a de novo assembly of R6837, which was produced by Spades v.3.8.0 [39] and annotated by Prokka v1.11 [40]. Reads from R6837 and R6838 were also aligned to the Mu50 genome (BA000017.4) to confirm the presence of SNPs identified by Werth *et al*., 2013 [32]. SAMtools/BCFtools 0.1.19 [41] detected SNPs from each reference alignment by considering base positions with a Phred score > = 20 and reads with a minimum mapping score of 20. We only analyzed SNPs at positions covered by a depth of 20 or more reads and where 95% of the reads supported the alternative allele. The presence of each potential SNP/Indel and its quality were confirmed in IGV v.2.3.78 [42] and mutations that occurred in highly variable regions (such as phage insertions) were discarded. Custom designed Python scripts associated mutations with coding or non-coding regions to identify changes potentially involved in DNS and to confirm the presence of previously recorded SNPs/Indels.

### Statistical analysis

Statistical analysis of all results was performed using GraphPad InStat Software Version 3.05. Analysis was carried out using one-way ANOVA with Tukey-Kramer post-test where appropriate and a *P* value of 0.05 or less was considered significant.

## Results

### DNS strains A6298, R6838 and JH4 have growth characteristics similar to their DAP-S parent strains, but exhibit increased MIC for DAP

**Fig 1:**
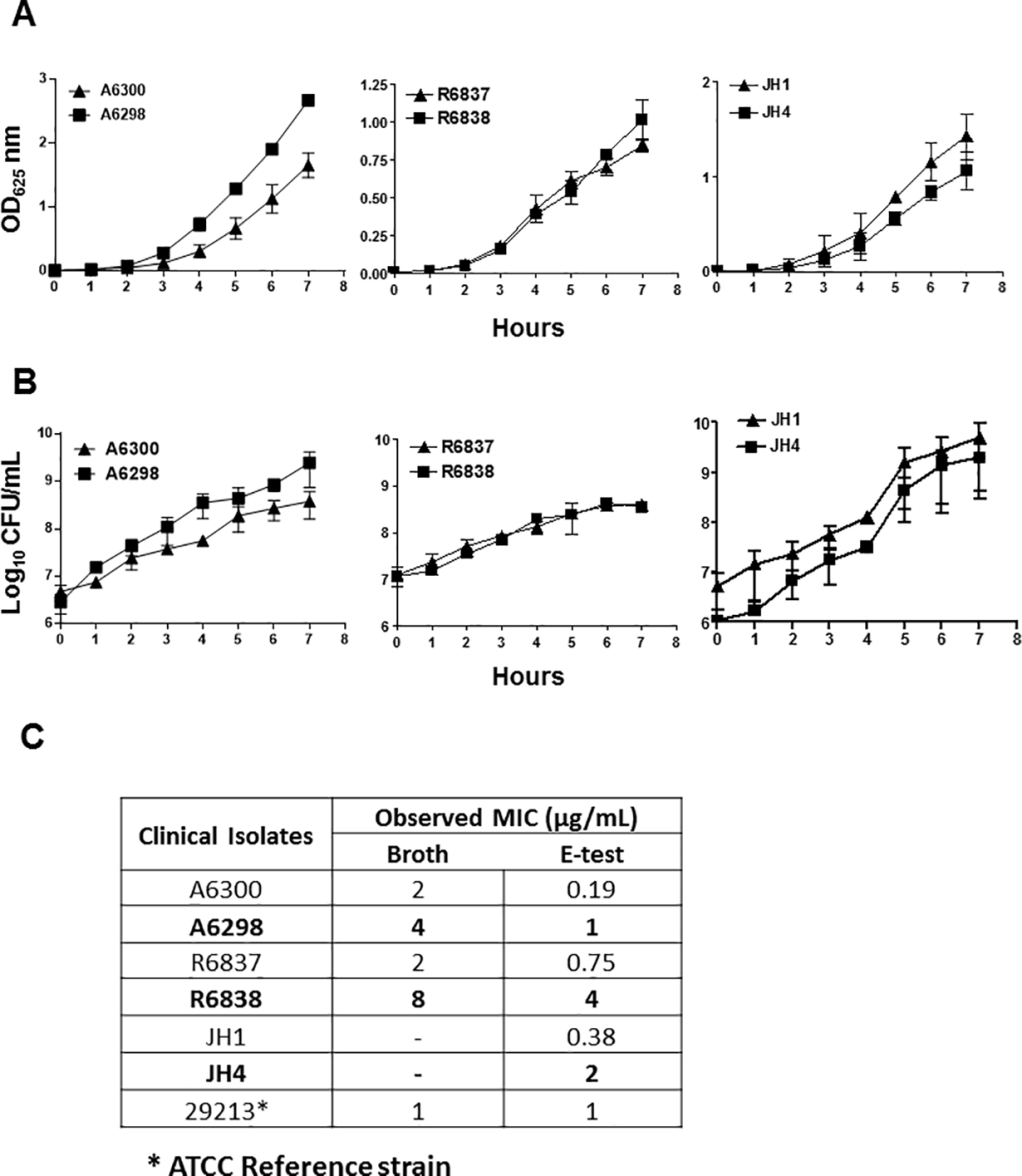
Growth characteristics and DAP susceptibility of DAP-S and DNS *S*. *aureus* strains. Bacterial growth of the indicated isogenic pairs of DAP-S and DNS *S*. *aureus* strains was monitored by measuring OD_600_ at 1-hour interval (A) and quantifying bacteria by plating on sheep blood agar plates. The colonies were counted and the results are expressed as Log_10_ CFU/mL (B). Minimum Inhibitory Concentrations (MICs) of DAP for isogenic DAP-S and DNS isogenic pairs as determined by microdilution method and E-test. The DNS *S*. *aureus* strains are represented in bold letters (C). The data shown are representative of two independent experiments conducted with similar results.

The three isogenic DAP-S/DNS pairs (A6300/A6298; R6837/R6838 and JH1/JH4) were characterized for their growth characteristics and susceptibility to DAP by generating bacterial growth curves, and colony counts at various time intervals. It was observed that growth of the isogenic DNS strains A6298, R6838 and JH4A was similar to their respective parent DAP-S strains A6300, R6837 and JH1 determined either by OD_600_ values or CFUs indicating that all the DAP-S/DNS isogenic pairs have identical growth patterns (Fig 1A and 1B).

The MICs of DAP for the isogenic DAP-S/DNS pairs was determined by broth microdilution assay and E-test. The DNS strains A6298, R6838 and JH4 exhibited 2-4-fold increase in MIC values as compared to their parent DAP-S strains A6300, R6837 and JH1. The E-test revealed a similar 2-5-fold increase in MIC for DAP for the DNS *S*. *aureus* strains (Fig 1C). Collectively, these results indicate that the DNS *S*. *aureus* strains have developed non-susceptibility to DAP however, their *in vitro* growth attributes remain unaltered.

### DAP-nonsusceptibility in *S*. *aureus* strains is associated with minor phenotypic changes

**Fig 2:**
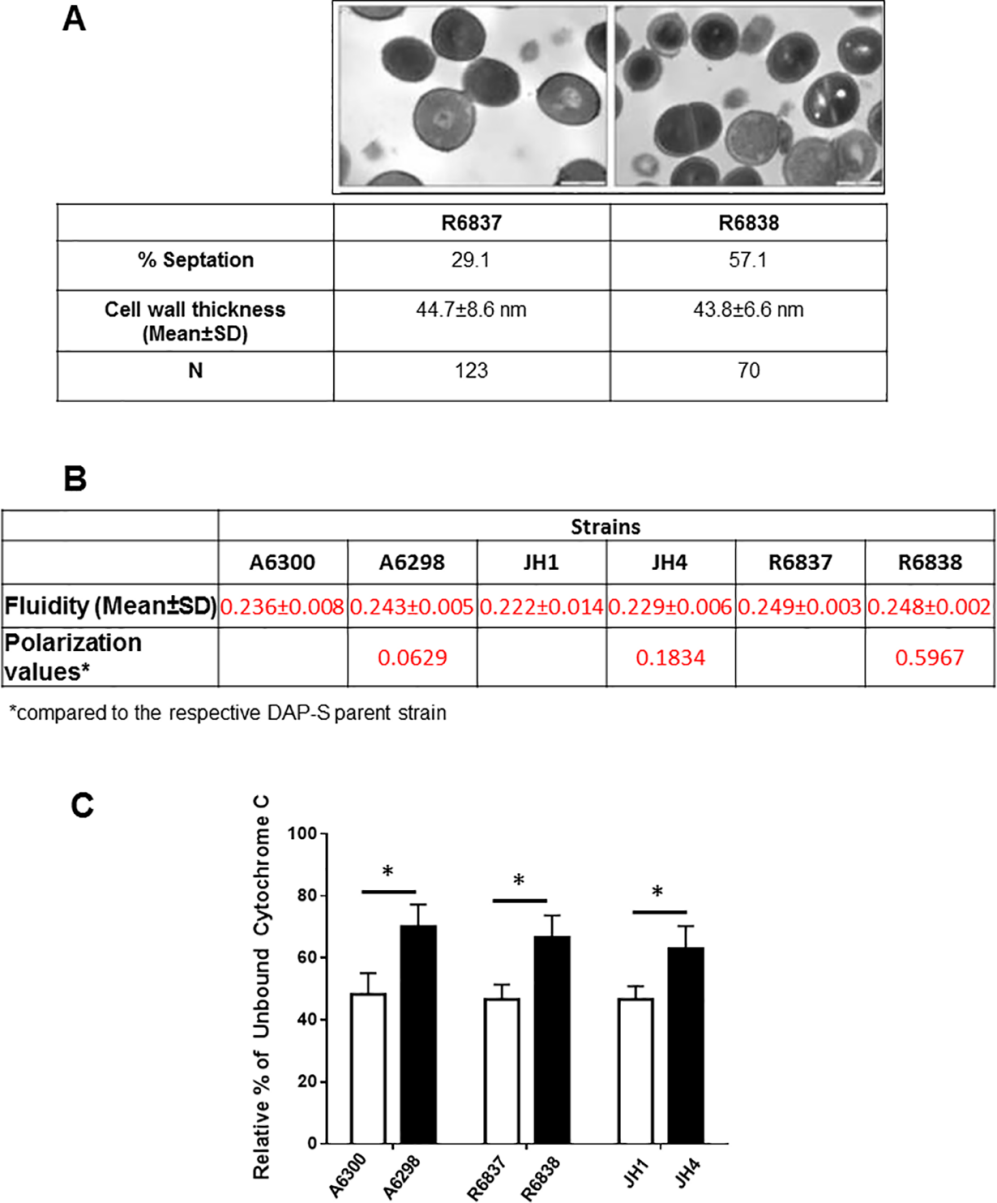
Phenotypic changes in DNS *S*. *aureus* strains. (A) Comparison of CW thickness and septation between DAP-S R6837 and DNS R6838 strains. TEM was used to measure the cell thickness and cell septation. N = total number of bacterial cells counted. (B) Membrane fluidity was calculated spectrofluorometrically and polarization index (*p*) were determined. Lower *p* value indicates higher membrane fluidity associated with DAP non-susceptibility. The differences in CW thickness (A) and polarization values (B) between DAP-S and DNS *S*. *aureus* strains are not statistically significant. (C) Determination of cell surface charge of the DAP-S and DNS *S*. *aureus* strains by cytochrome C binding assay. The data were analyzed by paired t test and P values were determined. *P<0.05.

Since CW thickness and cell morphology differences have often been associated with DNS, we next determined if the DAP-S parent strain R6837, and its non-susceptible counterpart R6838 exhibit any such differences. Cell morphology differences in septation and CW thickness between the isogenic DAP-S and DNS *S*. *aureus* isolates were examined by TEM. No difference in mean CW thickness was observed between DAP-S and DNS *S*. *aureus* strains (44.7±8.6 nm versus 43.8±6.6 nm, respectively). However, DNS R6838 strain displayed a significant increase in the percentage of cells with partial or complete septae. After overnight growth, 57.1% of R6838 cells showed septa formations as compared to 29.1% of the DAP-S R6837 isolate (Fig 2A). These results indicate that the septae formation is increased in DNS as compared to the DAP-S *S*. *aureus* strains.

Several reports have demonstrated an association between CM fluidity and DNS. The CM fluidity of all the three isogenic pairs of clinical isolates of DAP-S and DNS strains were determined spectrofluorometrically using a fluorescent probe according to a previously published protocol [35]. The isogenic DNS *S*. *aureus* strains A6298 and JH4 exhibited increased membrane fluidity as compared to their DAP-S parent strains (Fig 2B). However, these differences did not achieve statistical significance. We also determined the changes in the cell surface charge of the DAP-S and DNS *S*. *aureus* strains. The reduced binding of the cytochrome C indicates an enhanced positive charge on cell surface envelop [37]. It was observed that a higher percentage of unbound cytochrome C was detected in supernatants from DNS *S*. *aureus* A6298, R6838 and JH4 strains as compared to their DAP-S counterparts A6300, R6837 and JH1, respectively (Fig 2C). These results indicate an increased cell surface positivity in DNS as compared to the DAP-S strains. Collectively, these results demonstrate that the DNS in *S*. *aureus* is associated with increased septations and cell surface charge but not with enhanced CW thickness or CM fluidity.

### DNS *S*. *aureus* strains exhibit alterations in the expression of genes involved in maintenance of CM charge

**Fig 3:**
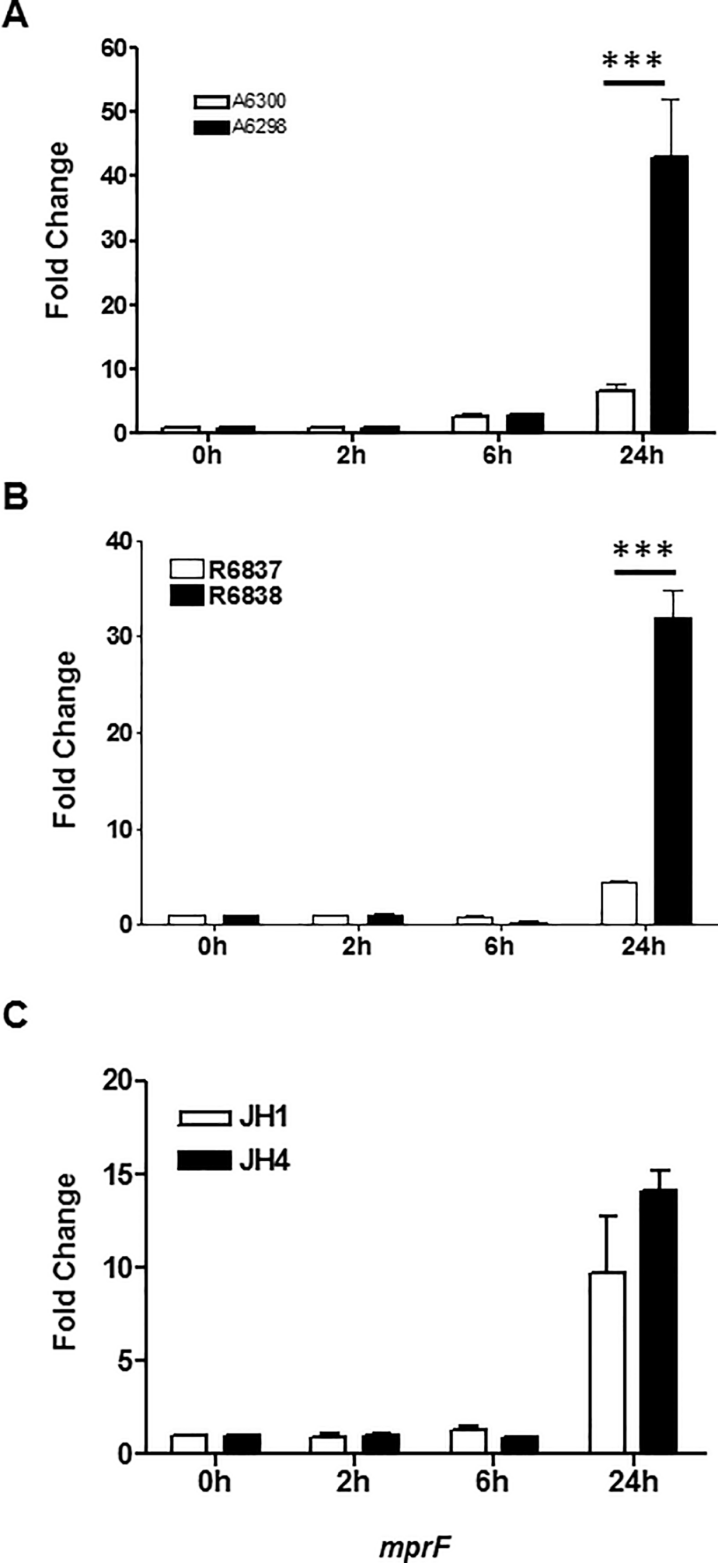
DNS *S*. *aureus* strain exhibits alterations in expression of genes involved in maintaining CM charge. Expression profile of *mprF* gene at the indicated times of bacterial growth by qRT-PCR. 16S rRNA was used as an internal control. Results are representative of at least two independent experiments. Statistical analysis was carried out using one-way ANOVA and a *P* value of 0.05 or less was considered significant. ***P<0.001.

One of the key genes involved in regulating the cell surface charge is the multipeptide resistance factor (*mprF)* gene. We investigated the expression of *mprF* gene in the three isogenic pairs of DAP-S and DNS *S*. *aureus* strains. Significantly elevated expression of the *mprF* gene was observed in DNS *S*. *aureus* strains A6298 and R6838 as compared to their isogenic parent DAP-S strains A6300 and R6837, respectively, after 24 hrs of growth. Although the *mprF* transcript levels were elevated after 24 hrs of growth in the DNS JH4 strain as compared to its isogenic DAP-S parent strain JH1, the statistical significance was not achieved (Fig 3).

**Fig 4:**
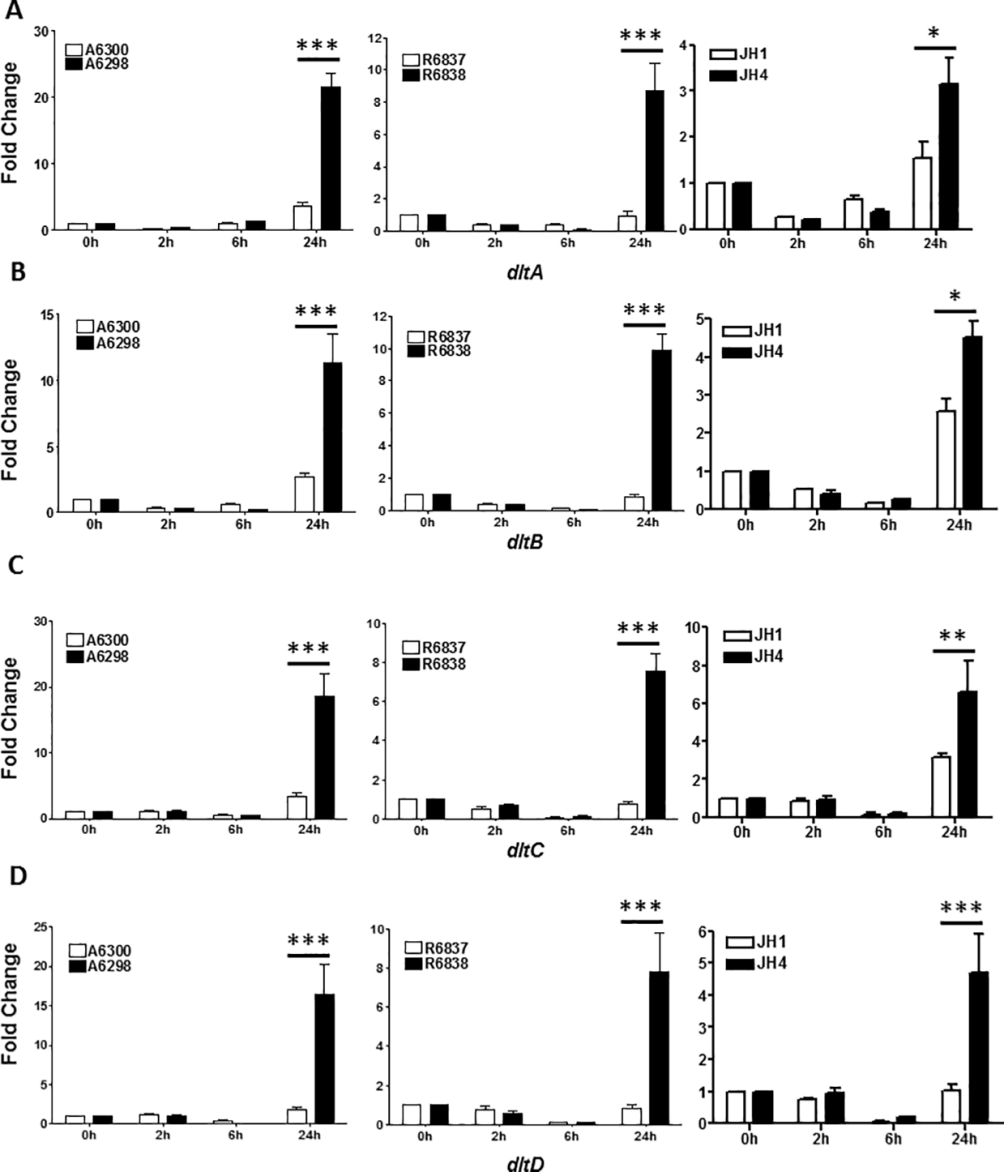
DNS *S*. *aureus* strains exhibit alterations in expression of genes involved in maintaining CM charge. Expression profile of (A) *dltA*; (B) *dltB;* (C) *dltC* and (D) *dltD* genes at the indicated times of bacterial growth by qRT-PCR. 16S rRNA was used as an internal control. Results are representative of at least two independent experiments performed. Statistical analysis was carried out using one-way ANOVA and a *P* value of 0.05 or less was considered significant. *P<0.05; **P<0.01; ***P<0.001.

Another group of genes that are involved in increasing the cell surface charge are those encoded on the *dltABCD* operon. Significantly elevated levels of *dltA*, *dltB*, *dltC* and *dltD* genes were observed in all DNS *S*. *aureus* strains as compared to their DAP-S parents after 24 hours of growth (Fig 4A–4D). These results indicate that the expression of genes involved in maintaining the cell surface charge are significantly upregulated in the DNS *S*. *aureus* strains.

### DNS *S*. *aureus* strains exhibit differential expression of genes involved in CW synthesis

**Fig 5:**
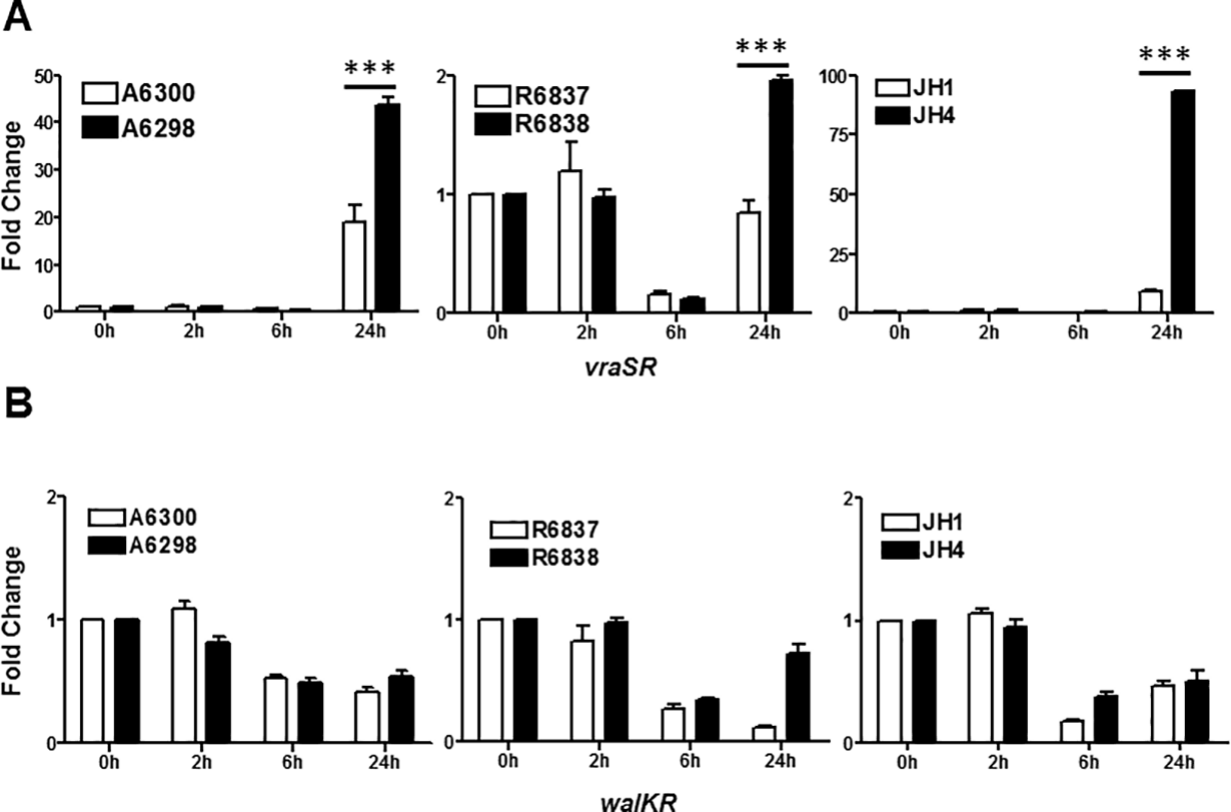
DNS *S*. *aureus* strains exhibit differential expression of genes involved in CW synthesis. Expression profile of (A) *vraSR* and (B) *walKR* genes at the indicated times of bacterial growth by qRT-PCR. 16S rRNA was used as an internal control. Results are representative of at least two independent experiments performed. Statistical analysis was carried out using one-way ANOVA and a *P* value of 0.05 or less was considered significant. ***P<0.001.

CW synthesis is mainly regulated by positive and negative feedback of specific genes. Two regulatory operons responsible for the synthesis of the CW are *vraSR* and *walK*. The transcription of CW synthesis-associated *vraSR* gene was analyzed by qRT-PCR. The transcript levels of *vraSR* gene were significantly upregulated in all DNS *S*. *aureus* strains as compared to the DAP-S parent strains after 24 hours of growth, but the extent of upregulation varied (Fig 5A).

*WalKR* (also known as *yycGF*) acts as the master controller of peptidoglycan metabolism, regulates fatty acid biosynthesis, and controls the activity of major autolysins genes *atl* and *lytM*. We found no significant alterations in the levels of *walKR* transcripts in DNS as compared to the DAP-S *S*. *aureus* strains after 2 hours of growth. However, the *walKR* transcript levels were down regulated in both DAP-S and DNS *S*. *aureus* strains after 6 and 24 hours of growth (Fig 5B).

**Fig 6:**
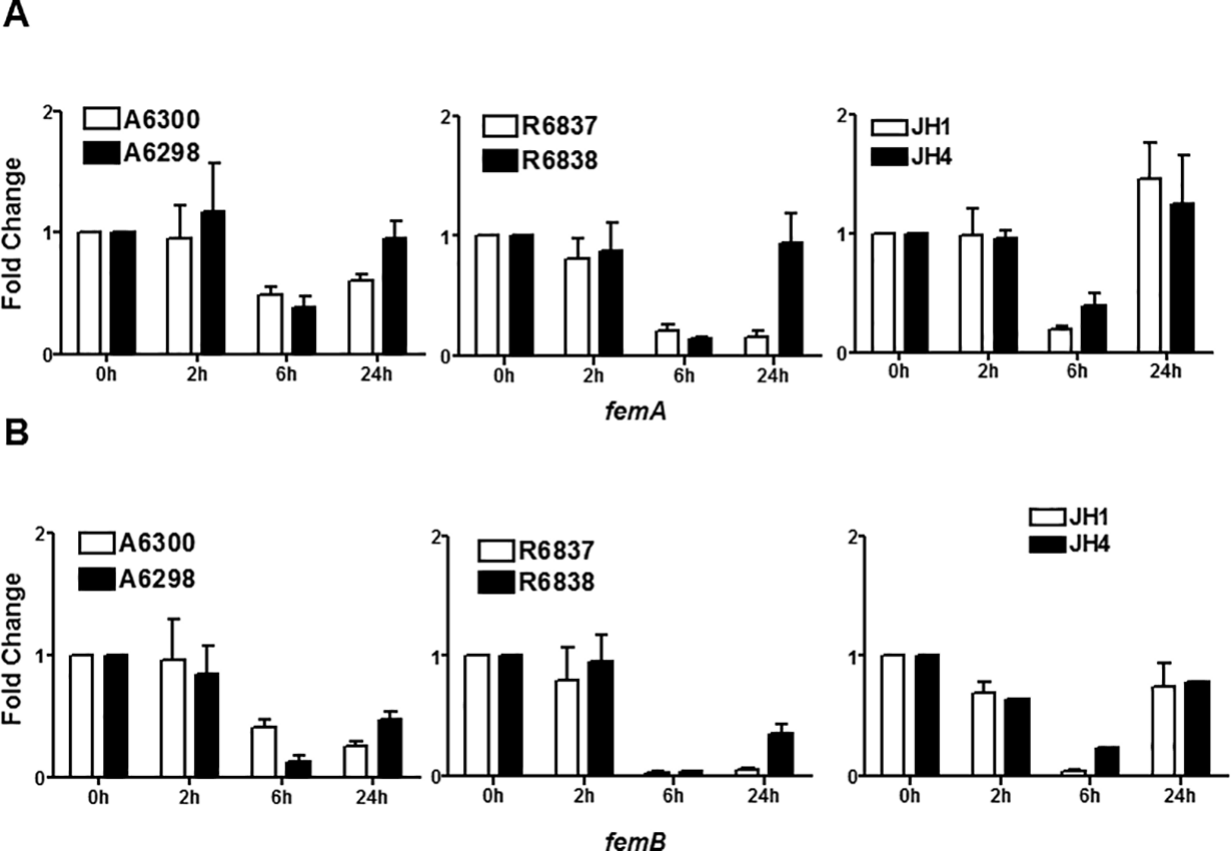
DAP-S or DNS *S*. *aureus* strains do not exhibit altered expression of *femA* and *femB* genes involved in CW synthesis. Expression profile of (A) *femA* and (B) *femB* genes at the indicated times of bacterial growth by qRT-PCR. 16S rRNA was used as an internal control. Results are representative of at least two independent experiments performed.

Additionally, factors essential for methicillin resistance (*fem)* also play a key role in developing resistance. We determined the expression profiles of *femA* and *femB* genes, which remained unaltered and no differences were observed between the DAP-S and DNS *S*. *aureus* strains (Fig 6A and 6B).

### Expression of penicillin binding proteins (PBPs) is altered in DNS *S*. *aureus* strains

**Fig 7:**
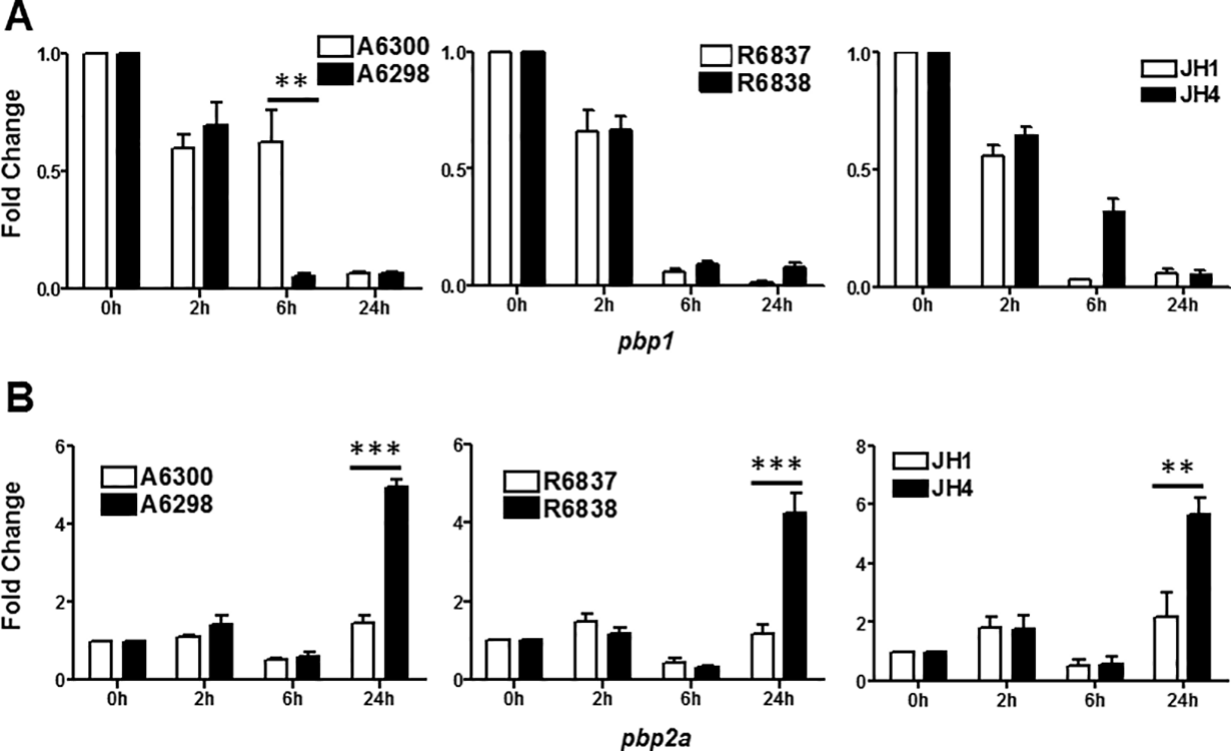
Expression of penicillin binding proteins (PBPs) is altered in DNS *S*. *aureus* strains. Expression profile of (A) *pbp1* and (B) *pbp2a* genes at the indicated times of bacterial growth by quantitative qRT-PCR. 16S rRNA was used as an internal control. Results are representative of at least two independent experiments. Statistical analysis was carried out using one-way ANOVA and a *P* value of 0.05 or less was considered significant. **P<0.01, ***P<0.001.

The antibacterial activity of beta-lactam antibiotics results from their covalent binding to the active sites of PBPs. Susceptible strains of *S*. *aureus* produce PBP1, PBP2, PBP3 and PBP4; however, MRSA strains produce abundant PBP2a protein, a transpeptidase to which most beta-lactam antibiotics cannot bind and therefore cannot exert their antibacterial actions. We investigated the expression of *pbp1* and *pbp2a* genes in DAP-S and DNS *S*. *aureus* strains. The expression levels of *pbp1* downregulated as the growth progressed in all the isogenic pairs of DAP-S and DNS *S*. *aureus* strains tested (Fig 7A). On the other hand, the DNS *S*. *aureus* strains (A6298, R6838 and JH4) revealed significantly elevated expression of the *pbp2a* gene as compared to the DAP-S strains (A6300, R6837 and JH1) after 24 hours of growth (Fig 7B). These results suggest that both DAP-S and DNS *S*. *aureus* strains maintain their resistance to beta-lactam antibiotics by reducing the expression of genes required for optimal attachment and binding. In addition, the DNS *S*. *aureus* strains upregulate *pbp2a* expression.

### DNS *S*. *aureus* strains exhibit upregulated expression of genes involved in autolysis

**Fig 8:**
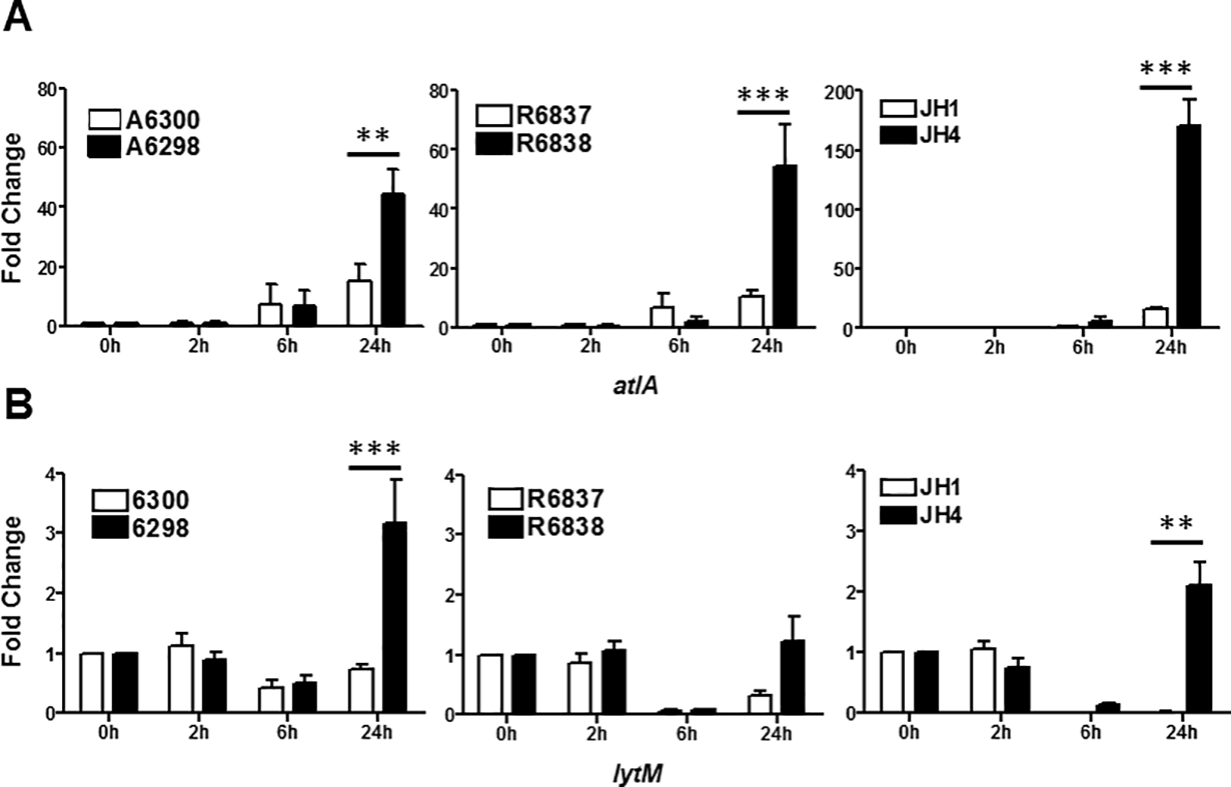
DNS *S*. *aureus* strains exhibit upregulated expression of genes involved in autolysis. Expression profile of (A) *atlA* and (B) *lytM* genes at the indicated times of bacterial growth by qRT-PCR. 16S rRNA was used as an internal control. Results are representative of at least two independent experiments performed. Statistical analysis was carried out using one-way ANOVA and a *P* value of 0.05 or less was considered significant. **P<0.01; ***P<0.001.

Differences in the expression of autolysis genes *atl* and *lyt*M have been reported to contribute to DAP non-susceptibility. We determined the expression of *atl* and *lyt*M genes in DAP-S and DNS *S*. *aureus* strains. We observed significantly higher transcript levels of *atl* gene in all DNS *S*. *aureus* strains after 24 hours of growth as compared to the DAP-S strains (Fig 8A). Similarly, we observed significantly higher transcript levels of *lytM* gene in the DNS strains A6298 and JH4, but not in R6838 as compared to the DAP-S strains A6300, JH1 and R6837 (Fig 8B).

### Mutations observed in DNS *S*. *aureus* strains

Whole genome sequencing was performed to identify mutations, which may contribute to DAP-nonsusceptibility in *S*. *aureus* strains as compared to their DAP-S isogenic counterparts. All single nucleotide polymorphisms (SNPs) detected by aligning the DNS strains to their DAP-S isogenic mate were also identified by isogenic pair alignments to the reference *S*. *aureus* strains N315 and Mu50, indicating a consistency of our results regardless of the reference genome employed. We identified most mutations that were previously reported for JH4, JH5, R6838, and A6298 strains [32, 43, 44], further confirming the quality of our results. Additionally, we detected novel mutations not identified in the previous studies, including two nonsynonymous mutations within the 50S ribosomal L3 protein (gene SA2047) in both the JH4 and JH5 strains, two substitutions upstream of lysine decarboxylase gene (SA0439) in JH4, and a nonsynonomous substitution in the *vraG* gene of JH5. However, we did not detect a nonsynonomous mutation reported in the SA20124 gene of A6298 [44] or a 9 base pair deletion identified in the SA1249 gene of JH5 strains [34]. Comparisons of the mutations from DNS strains for identification of genetic markers for vancomycin and DNS did not reveal any common mutations except for the JH4 strain (Table 2).

**Table 2:** Mutational differences between DNS and DAP-S isogenic strain pairs identified by whole genome sequencing.

Isogenic pair (DAP-S/DNS)	Mutation position	Mutation type	Locus abbreviation/Gene name	Locus description	Notes
**A6300/A6298**	**649970**	**Synonymous F147F**^**a**^	**SA0556**	**Conserved hypothetical protein**	**[44]**
A6300/A6298	670496	Synonymous G174G	SA0578	NADH dehydrogenase	
A6300/A6298	802280	Noncoding	SA0704	Conserved hypothetical protein	
**A6300/A6298**	**1052756**	**Nonsynonymous V353A**^**a**^	**purD/SA0926**	**Phosphoribosylamine—glycine ligase PurD**	**[44]**
**A6300/A6298**	**1604155**	**Nonsyonymous E66K**^**a**^	**SA1398**	**Diacylglycerol kinase**	**[44]**
**A6300/A6298**	**1608805**	**Frameshift Tx1 deletion**^**a**^^**,**^^**b**^	**rpsU/SA1404**	**30S ribosomal protein S21**	**[44]**
**A6300/A6298**	**1700172**	**Nonsynonymous G48D**^**a**^	**hemL/SA1491**	**Gutamate-1-semialdehyde 2%2C1-aminomutase**	**[44]**
**A6300/A6298**	**2792195**	**Nonsynonymous N83S**^**a**^	**drp35/SA2480**	**Drp35**	**[44]**
**R6837/R6838**	**1364633**	**Nonsynonymous L341S**^**a**^	**mprF/SA1193**	**Oxacillin resistance-related MprF protein**	**Same as Mu50 position 1440962 in gene SAV1360 [32]**
**R6837/R6838**	**1532504**	**Synonymous I34I**^**a**^	**srrB/SA1322**	**Staphylococcal respiratory response protein SrrB**	**Same as Mu50 position 1608916 in gene SAV1491 [32]**
**R6837/R6838**	**1882832**	**Noncoding**^**a**^	**SA1649**	**Conserved hypothetical protein**	**Same as Mu50 position 1960627 [32]**
R6837/R6838	2009715	Noncoding	SA1759	Lytic enzyme	R6838 reversion to N315 reference
**R6837/R6838**	**2378757**	**Nonsynonymous D31E**^**a**^	**SA2115**	**Transcriptional regulator**	**Same as Mu50 position 2448257 in gene SAV2324, R6838 reversion to N315 reference [32]**
**R6837/R6838**	**2521688**	**Nonsynonymous A151V**^**a**^	**SA2244**	**Endo-1,4-beta-glucanase**	**Same as Mu50 position 2592882 in gene SAV2455 [32]**
**JH1/JH4, JH1/JH5**	**27546**	**stop codon at AA 36**^**a**^	**SA0019**	**Conserved hypothetical protein**	**Originally identified in JH6 [34]**
JH1/JH4	511808	Noncoding	SA0439	Lysine decarboxylase	
JH1/JH4	511809	Noncoding	SA0439	Lysine decarboxylase	
**JH1/JH4, JH1/JH5**	**581030**	**Nonsynonymous D471Y**^**a**^	**rpoB/SA0500**	**RNA polymerase beta chain**	**Originally identified in JH2 [34]**
**JH1/JH4, JH1/JH5**	**581036**	**Nonsynonymous A473S**^**a**^	**rpoB/SA0500**	**RNA polymerase beta chain**	**Originally identified in JH2 [34]**
**JH1/JH4, JH1/JH5**	**581048**	**Nonsynonymous A477S**^**a**^	**rpoB/SA0500**	**RNA polymerase beta chain**	**Originally identified in JH2 [34]**
**JH1/JH4, JH1/JH5**	**581053**	**Nonsynonymous E478D**^**a**^	**rpoB/SA0500**	**RNA polymerase beta chain**	**Originally identified in JH2 [34]**
**JH1/JH4, JH1/JH5**	**585867**	**Nonsynonymous E854K**^**a**^	**rpoC/SA0501**	**RNA polymerase beta-prime chain**	**Originally identified in JH2 [34]**
**JH1/JH4, JH1/JH5**	**674706**	**Synonymous S30S**^**a**^	**SA0582**	**Na+/H+ antiporter**	**Originally identified in JH6 [34]**
JH1/JH4	792565	Nonsynonymous Y171C	SA0694	Conserved hypothetical protein	
JH1/JH4	810100	Synonymous I242I	prfB/SA0709	Peptide chain release factor 2	
JH1/JH4	810711	Noncoding	SA0710	Conserved hypothetical protein	
**JH1/JH4, JH1/JH5**	**1110065**	**Nonsynonymous A84V**^**a**^	**isdE/SA0980**	**Ferrichrome ABC transporter**	**Originally identified in JH6 [34]**
**JH1/JH4, JH1/JH5**	**1282852**	**Nonsynonymous D296Y**^**a**^	**SA1129**	**Conserved hypothetical protein**	**Originally identified in JH2 [34]**
JH1/JH4	1408780	Noncoding	CspA/SA1234	Major cold shock protein CspA	
JH1/JH4	1583224	Nonsynonymous L46P	SA1378	Conserved hypothetical protein	
**JH1/JH4, JH1/JH5**	**1893513**	**Frameshift Ax7 deletion**^**a**^^**,**^^**b**^	**prsA/SA1659**	**Peptidyl-prolyl cis/trans isomerase**	**Originally identified in JH6 [34]**
**JH1/JH4, JH1/JH5**	**1948612**	**Nonsynonymous H164R**^**a**^	**SA1702**	**Conserved hypothetical protein**	**Originally identified in JH2 [34]**
JH1/JH4, JH1/JH5	2307851	Nonsynonymous G152D	rplC/SA2047	50S ribosomal protein L3	
JH1/JH4, JH1/JH5	2307876	Nonsynonymous G144S	rplC/SA2047	50S ribosomal protein L3	
**JH1/JH4, JH1/JH5**	**2354954**	**Nonsynonymous A94T**^**a**^	**SA2094**	**Na+/H+ antiporter**	**Originally identified in JH6 [34]**
**JH1/JH4, JH1/JH5**	**2391175**	**Noncoding**^**a**^	**SA2126**	**Hypothetical protein**	**Originally identified in JH6 [34]**
**JH1/JH4, JH1/JH5**	**2604820**	**Synonymous D168D**^**a**^	**SA2320**	**Regulatory protein pfoR**	**Originally identified in JH6 [34]**
JH1/JH4	2630178	Synonymous G242G	rocA/SA2341	1-pyrroline-5-carboxylate dehydrogenase	
JH1/JH5	85776	synonymous Q64Q	SA0077	Serine/threonine protein kinase	
JH1/JH5	180296	Nonsynonymous E101G	capM/SA0156	Capsular polysaccharide synthesis enzyme Cap5M	
JH1/JH5	255175	Nonsynonymous D197G	SA0215	Two-component response regulator	
JH1/JH5	396355	Nonsynonymous A257V	SA0339	ABC transporter ATP-binding protein	
JH1/JH5	448576	Synonymous S221S	set12/SA0388	Exotoxin 12	
JH1/JH5	484526	Stop codon at AA 254	SA0422	Lactococcal lipoprotein	
JH1/JH5	621945	Noncoding	nagB/SA0527	Glucosamine-6-phosphate isomerase	
JH1/JH5	712573	Nonsynonymous A580V	vraG/SA0617	ABC transporter permease	
JH1/JH5	751554	Noncoding	SA0657	Hemolysin	
JH1/JH5	1005831	Nonsynonymous C147Y	SA0885	Hypothetical protein	
JH1/JH5	1893057	Nonsynonymous E140Q	prsA/SA1659	Peptidyl-prolyl cis/trans isomerase	
JH1/JH5	1954969	Nonsynonymous D48H	SA1708	UDP-N-acetylmuramyl tripeptide synthetase	
JH1/JH5	2368738	Nonsynonymous H177R	SA2105	Conserved hypothetical protein	
JH1/JH5	2382895	Synonymous P247P	SA2119	Dehydrogenase	
JH1/JH5	2393142	Nonsynonymous M33T	SA2127	Ribose 5-phosphate isomerase (rpi)	

^**a**^Mutations identified in previous publications are shown in bold letters.

^b^N x number = nucleotide x number of repeats

## Discussion

In the recent past, DNS *S*. *aureus* strains resulting from treatment failures have been increasingly reported [45]. Cases of refractory bacteremia persisting for days or even weeks treated initially with vancomycin and subsequently with DAP, in association with host innate immune system and antimicrobial therapy provide a selective pressure for emergence of DNS *S*. *aureus*. Unlike phenotypic changes associated with the DNS *S*. *aureus* strains, the genotypic changes, especially the kinetics of alterations in the expression of genes responsible for causing DAP-nonsusceptibility are not well understood. In this study, we investigated the expression profiles of genes involved in CM charge (*mprF*, *dltABCD*), CW synthesis (*femA*, *femB*, *vraSR*, and *walKR*); autolysis (*atl*, *lytM*); and penicillin binding (*pbp1* and *pbp2a*) in three isogenic pairs of DAP-S and DNS *S*. *aureus* strains.

It has been postulated that in DNS *S*. *aureus* strains a “charge-repulsive *milieu*” is responsible for repulsion/non-binding of DAP-calcium complex [46]. Reports suggest that the *mprF* and *dlt* genes of *S*. *aureus* act by increasing the net surface positive charge [11, 19]. The *mprF* gene alters the net surface charge by lysinylating the membrane PG to generate a positively-charged L-PG which ultimately translocate to the outer membrane leaflet. Unlike *mprF*, the *dltABCD* operon acts by D-alanylating CW teichoic acids creating a greater net positive charge, thereby reducing the access of DAP to the CM [4, 19, 23]. The increased expression of *mprF* gene has been reported to be associated with enhanced DNS [15, 22, 47]. *S*. *aureus* strains exhibiting gain-of-function *mprF* mutations have increased positive cell surface charge and reduced capacity to bind DAP [11]. Consistent with these findings, we observed significantly elevated expression of the *mprF* and *dltABCD* operon genes in DNS *S*. *aureus* strains indicating that increased expression of *mprF*, and *dlt* operon genes may cause an increased positive cell surface charge and reduced binding of DAP, thereby contributing to non-susceptibility. These findings were also corroborated by an enhanced positive charge in the DNS *S*. *aureus* strains in cytochrome C binding assays. The enhanced expression of *mprF* gene DNS *S*. *aureus* strains is akin to the “gain in *mprF* function” associated with mutations in *mprF* gene [48].

The “membrane order hypothesis” associates phenotypic adaptations that include alterations in the composition of CM fatty acids, and enhanced CW teichoic acids and peptidoglycan synthesis resulting in increased CM fluidity [21, 49] and CW thickness [4, 50], respectively. Consistent with these observations, we demonstrate an increased expression of *vraSR* gene in DNS as compared to DAP-S *S*. *aureus* strains. Bertsche *et al*., 2013 demonstrated a correlation between thickened CW and increased cell surface charge [4]. CW thickening, however, has been shown to be sufficient but not necessary for development of DNS [15, 17, 51].

*VraSR* and *walKR/yyCGF/vicRK* are two-component regulatory systems which regulate CW biosynthesis [52]. Over expression of *vraSR* in DAP-S strains is associated with increased DAP-nonsusceptibility [47]. Consistent with previous reports [15, 17], despite an upregulated expression of *vraSR* in the DNS *S*. *aureus* strains, the CW thickness remained unchanged in DAP-S and DNS isogenic strains (**Fig 2A**). *WalKR* is the master controller of peptidoglycan metabolism and its depletion leads to CW thickening and defects in cell division due to its role in coordination of CW metabolism and cell division [53]. *WalKR* also controls the activity of major autolysin genes *atl* and *lytM* [54]. The *atl* gene encodes a bi-functional enzyme with an amidase and a glucosaminidase domain that represents the most predominant peptidoglycan hydrolase of *S*. *aureus*; whereas the *lytM* gene encodes a Gly-Gly endopeptidase. Song *et al*., 2013 demonstrated a lower rate of autolysis in the DNS strain [55]. It is worth noting that upregulation of the autolytic genes *atlA* and *lytM* in the DNS *S*. *aureus* strains indicate an autolytic cell death unlike that observed for DAP-S counterparts. Collectively, these results demonstrate that the major mechanism of DNS in *S*. *aureus* strains appears to be due to the alteration of expression of genes involved in maintenance of cell surface charge rather than those involved in increasing the CW thickness.

Another important finding from the study was differential expression of PBP genes in DAP-S and DNS *S*. *aureus* strains. Thus, it was interesting to observe that the DNS strains had significantly elevated expression of *pbp2a* gene as compared to the DAP-S strain even in the drug free conditions of our study. On the other hand, the expression of *pbp1* was significantly downregulated in both DAP-S as well as DNS *S*. *aureus* strains. PBP1 is located in the septum and plays an important role in cell division of *S*. *aureus* [56]. Our analysis of cells by TEM found a greater percentage of cells with septum formation in DNS R6838 strain. Since expression of PBP1 was downregulated over time in both DAP-S and DNS strains, it is our hypothesis that PBP1 may be important for the completion of cell division, but perhaps not required for septa initiation. Thus, reduced PBP1 may leave partially initiated septae that do not result in cell division.

Several reports suggest that genetic mutations are important for conferring DNS [55, 57]. Mutations in the CW synthesis genes (*mprF*, *agrC*), RNA polymerases (*rplV*, *rplC*), two component systems (*wal*KR/*vic*R), and proteases (*clpP*) have been associated with antibiotic resistance [58]. In our study, all DNS clinical strains sequenced had non-synonymous mutations in genes that could ultimately alter CM charge, CW thickness and CW composition. The whole genome sequencing analysis revealed several new SNPs in DNS strains, including two nonsynonymous mutations within the 50S ribosomal L3 protein (gene SA2047) of both the JH4 and JH5 strains, two substitutions in the JH4 strain upstream of a lysine decarboxylase (SA0439) gene which potentially interacts with BlaR1; the integral membrane protein that confers β-lactam resistance [59], and a nonsynonymous substitution in the *vraG* gene of JH5 strain. The *vraG* gene is part of the *vraFG* locus, which is an ABC transporter-dependent efflux pump and is located downstream of a two-component regulatory system, *GraRS*. Co-transcription of both the *vraG* and *graR* is required for the expression of *mprF* and *dlt* genes which play an important role in maintaining net positive surface charge and resistance to cationic antimicrobial peptides [22]. Furthermore, Mwangi *et al*., 2013 [43] observed a progression of mutations from strains JH1-JH9 that correlated with increasing antibiotic resistance, we detected a majority of the mutations that were reported for JH6 were present in JH4 and JH5 strains. We believe that this is possibly due to the increased depth of the sequence coverage provided by Illumina sequencing and suggests that mutations do not necessarily occur in a sequential order. Mutations in *yycH*, which controls CW and CM turnover through the essential two component WalKR system, were observed in both the DNS JH4 and JH5 strains of *S*. *aureus* [43, 60, 61]. Mutations in *rpo* genes were also observed in JH4 and JH5 isolates. While mutations in *rpo* genes are not thought to directly contribute to antibiotic resistance, they may adjust the global transcriptional profile and develop DNS phenotype [62, 63]. DNS isolate A6298 was additionally found to have a mutation in *dgkA* gene, which encodes undecaprenol kinase (UDPK). UDPK recycles undecaprenol by phosphorylation to form undecaprenol phosphate (bactoprenol) which is a precursor of lipid carriers involved in CW biogenesis [64, 65]. Another DNS isolate R6838 harbored a mutation in the phosphatidylglycerol lysyltransferase (*mprF*), which adds a positively charged lysine residue to PG and flips L-PG into the outer membrane. The *mprF* mutations in clinical DNS isolates including the mutations that corresponds to L341S mutation in R6838 isolate observed in this study [66]. This mutation is in the bifunctional domain of *mprF*, which could impact L-PG content in the CM, changing the net membrane charge and resulting in DNS in combination with expression changes [14].

Our DNA sequence analysis also revealed that resistance to vancomycin and DAP evolves in multiple ways as none of the resistant strains showed identical mutations except for JH4 and JH5 which are derived from the same parent. Thus, our results further establish the notion that multiple genetic mechanisms are involved in the development of DNS in *S*. *aureus*. It is noteworthy to mention that the manner in which the development of DAP resistance occurred in DNS *S*. *aureus* strains varied. The JH4-JH5 and A6298 DNS strains were derived from patients that were never treated with DAP. This resistance has been reported to occur in response to host’s cationic antimicrobial peptides which may provide an endogenous selection pressure for the development of DAP-nonsusceptibility [6]. However, the DAP-nonsusceptibility in R6838 strain was observed while the patient was on DAP therapy following treatment failure with vancomycin. These observations indicate that both the endogenous and exogenous antimicrobial selection pressure may play an important role in the development of DNS in *S*. *aureus*.

Taken together, this study unravels some of the complex molecular changes involved in the development of DNS and demonstrates that differences in gene expression profiles and mutational differences in the DNS *S*. *aureus* strains can serve as markers for differentiation of DAP-S and DNS *S*. *aureus* in clinical settings.
